# Norovirus Escape from Broadly Neutralizing Antibodies Is Limited to Allostery-Like Mechanisms

**DOI:** 10.1128/mSphere.00334-17

**Published:** 2017-10-18

**Authors:** Abimbola O. Kolawole, Hong Q. Smith, Sophia A. Svoboda, Madeline S. Lewis, Michael B. Sherman, Gillian C. Lynch, B. Montgomery Pettitt, Thomas J. Smith, Christiane E. Wobus

**Affiliations:** a Department of Microbiology and Immunology, University of Michigan Medical School, Ann Arbor, Michigan, USA; b Department of Biochemistry and Molecular Biology, University of Texas Medical Branch at Galveston, Galveston, Texas, USA; Erasmus MC

**Keywords:** antibody, neutralization, noroviruses, protein structure-function

## Abstract

Ideal antiviral vaccines elicit antibodies (Abs) with broad strain recognition that bind to regions that are difficult to mutate for escape. Using 10 murine norovirus (MNV) strains and 5 human norovirus (HuNoV) virus-like particles (VLPs), we identified monoclonal antibody (MAb) 2D3, which broadly neutralized all MNV strains tested. Importantly, escape mutants corresponding to this antibody were very slow to develop and were distal to those raised against our previously studied antibody, A6.2. To understand the atomic details of 2D3 neutralization, we determined the cryo-electron microscopy (cryo-EM) structure of the 2D3/MNV1 complex. Interestingly, 2D3 binds to the top of the P domain, very close to where A6.2 binds, but the only escape mutations identified to date fall well outside the contact regions of both 2D3 and A6.2. To determine how mutations in distal residues could block antibody binding, we used molecular dynamics flexible fitting simulations of the atomic structures placed into the density map to examine the 2D3/MNV1 complex and these mutations. Our findings suggest that the escape mutant, V339I, may stabilize a salt bridge network at the P-domain dimer interface that, in an allostery-like manner, affects the conformational relaxation of the P domain and the efficiency of binding. They further highlight the unusual antigenic surface bound by MAb 2D3, one which elicits cross-reactive antibodies but which the virus is unable to alter to escape neutralization. These results may be leveraged to generate norovirus (NoV) vaccines containing broadly neutralizing antibodies.

**IMPORTANCE** The simplest and most common way for viruses to escape antibody neutralization is by mutating residues that are essential for antibody binding. Escape mutations are strongly selected for by their effect on viral fitness, which is most often related to issues of protein folding, particle assembly, and capsid function. The studies presented here demonstrated that a broadly neutralizing antibody to mouse norovirus binds to an exposed surface but that the only escape mutants that arose were distal to the antibody binding surface. To understand this finding, we performed an *in silico* analysis that suggested that those escape mutations blocked antibody binding by affecting structural plasticity. This kind of antigenic region—one that gives rise to broadly neutralizing antibodies but that the virus finds difficult to escape from—is therefore ideal for vaccine development.

## Abstract (precis)

The simplest and most common way for viruses to escape antibody neutralization is by mutating residues that are essential for antibody binding. Escape mutations are strongly selected for by their effect on viral fitness, which is most often related to issues of protein folding, particle assembly, and capsid function. The studies presented here demonstrated that a broadly neutralizing antibody to mouse norovirus binds to an exposed surface but that the only escape mutants that arose were distal to the antibody binding surface. To understand this finding, we performed an *in silico* analysis that suggested that those escape mutations blocked antibody binding by affecting structural plasticity. This kind of antigenic region—one that gives rise to broadly neutralizing antibodies but that the virus finds difficult to escape from—is therefore ideal for vaccine development.

## INTRODUCTION

There have been a number of recent successes in isolating broadly neutralizing antibodies (bnAbs) to different viruses such as human immunodeficiency virus (HIV) and influenza virus (1, 2). Vaccines eliciting such antibodies, called universal vaccines, are highly desirable to reduce the need for reformulation, e.g., after each influenza virus pandemic outbreak, and to provide protection against viruses with extensive genetic diversity (3–5). Human norovirus (HuNoV) is another example of a virus that causes recurrent pandemics and exhibits extensive genetic diversity but without an approved vaccine (6, 7). This is in part due to the historical lack of a cell culture system (8–10) and a small-animal model (11) that has prevented the identification of neutralizing antibodies. Monoclonal antibodies (MAb) have been identified that block binding of HuNoV to the carbohydrate attachment receptor mostly in a strain-specific manner, including the pandemic-causing genogroup II, genotype 4 (GII.4) HuNoV strains (12–14). However, no infection-neutralizing antibodies have yet been described for HuNoV strains in spite of the very recent development of HuNoV culture systems. With biology similar to that of HuNoV and the availability of robust reverse genetics, cell culture, and mouse models, murine norovirus (MNV) is a versatile tool to study NoV biology, including the mechanisms of NoV antibody neutralization (15–21). Therefore, understanding the mechanism by which anti-MNV MAbs interact with and neutralize diverse MNV strains may facilitate development of bnAbs for HuNoV.

**FIG 1:**
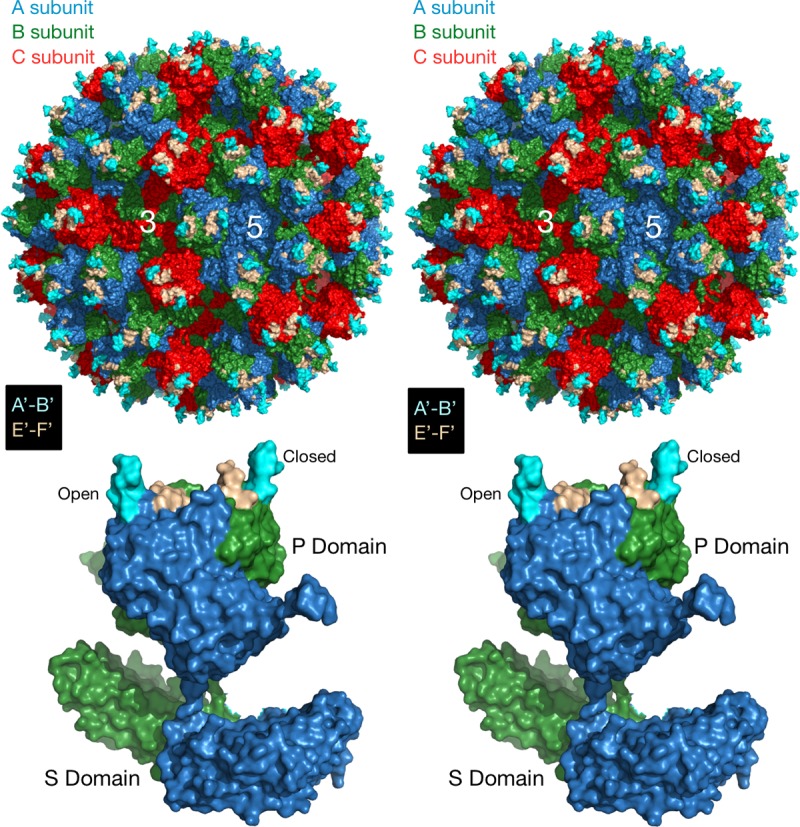
Structure of MNV. The top stereo diagram shows the surface of the MNV T=3 capsid, with the A, B, and C subunits shown in blue, green, and red, respectively. The A′-B′ and E′-F′ loops are highlighted in cyan and wheat colors, respectively. The 3-fold and 5-fold axes are noted. The bottom stereo figure shows a side view of the A and B subunits. The P and S domains are noted and the A′-B′ and E′-F′ loops are noted as described above. Note how the P domains float above the S domain. Also shown are the open and closed conformations of the A′-B′/E′-F′ loops.

The NoV capsid protein is composed of a highly conserved shell (S) and protruding (P) domain (22–25) (Fig. 1). The P domain contains binding sites for receptors (26, 27) and neutralizing antibodies (28–30). We previously isolated and characterized a panel of monoclonal antibodies (MAbs) against MNV1 (15, 20, 30, 31); of those, MAb 5C4.10 (called 5C4 here) was mapped to the S domain of the capsid of MNV1-CW3 (referred to as MNV1 here) but did not neutralize MNV1, while the MNV1 neutralizing MAb A6.2 and MAbs 2D3.7 and 4F9.4 (here called 2D3 and 4F9, respectively) bound to the MNV1 P domain (15, 25, 30, 31). Specifically, MAb A6.2 binds to the top of the highly flexible E′-F′ loop of the P domain when it is in the "open" (A) conformation but not when it is in the "closed" (B) conformation (30). While the escape mutants corresponding to MAb A6.2 are in the antibody contact region and most likely sterically block antibody binding, some of our previously generated site-directed mutations (e.g., L386F and A382R/K) likely block antibody binding by affecting the conformation of the E′-F′ loop (15). We previously demonstrated that MAbs 2D3 and 4F9 bind MNV1 with higher affinity than MAb A6.2 and that escape mutations arise more slowly (31). However, the mechanism of virus escape from these two MAbs is not yet understood, since the identified neutralizing escape mutations, V339I and D348E, lie deep within the P domain (31) (also see Fig. 6). Closing this knowledge gap was the goal of this study. Given that at least one HuNoV MAb (against Norwalk virus [NV]) also binds to the E′-F′ loop (32) and that HuNoV and MNV share close structural similarities of the virus capsid, it is likely that mechanisms identified with MNV also extend to HuNoV neutralization escape.

The experiments reported here were performed to analyze the binding and neutralization activities of MAbs 5C4, A6.2, 2D3, and 4F9 against 10 MNV strains and five HuNoV virus-like particles (VLPs) to determine the molecular basis of neutralization and escape. Although MAb 5C4 bound to all MNV strains and HuNoV VLPs tested, none of the MNV strains were neutralized by this MAb. In contrast, MAbs A6.2, 4F9, and 2D3 bound all tested MNV strains and exhibited neutralization activities that ranged from narrow (A6.2) to broad (2D3). Since MAb 2D3 neutralized all 10 MNV strains, cryo-electron microscopy (cryo-EM) image reconstruction was used to determine the structure of the MNV1/2D3 complex. Surprisingly, it was similar to that of the A6.2/MNV1 structure (25), in spite of the fact that escape mutations corresponding to MAb A6.2 do not affect MAb 2D3 binding (31). Although the only identified escape mutations corresponding to MAb 2D3 are distal to the antibody contact region, molecular dynamics flexible fitting simulations suggested that these mutations cause long-range structural changes in the P domain via a novel mechanism involving a series of salt bridges between the P domain dimers. These data highlighted that the region encompassing the escape mutations is structurally conserved among MNV strains and thus may represent an ideal vaccine epitope for HuNoVs.

## RESULTS

### Monoclonal antibody 5C4 binds murine noroviruses and human norovirus VLPs.

**FIG 2:**
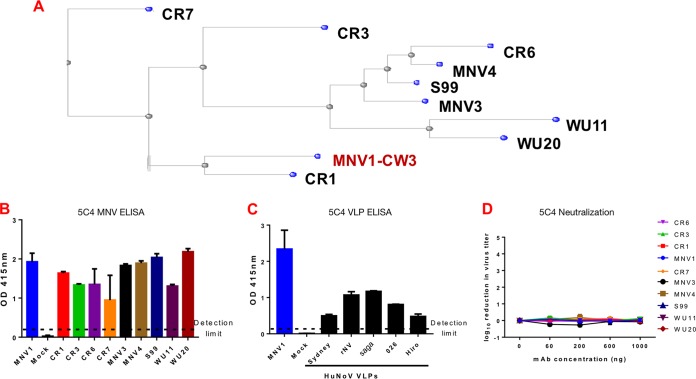
5C4 monoclonal antibody (mAb) binds to noroviruses. (A) Neighbor-joining phylogenetic tree of 10 murine norovirus (MNV) strains using the 541-amino-acid capsid protein. (B and C) Plates were coated with MNV strains (B) or human norovirus (HuNoV) VLPs (C) and tested for reactivity to 1 μg per well of MAb 5C4. MNV1 and mock lysate were used as positive and negative controls, respectively. The dashed line represents twice the absorbance values at 415 nm (*A*_415_) for the negative control and indicates the threshold for a positive signal. OD, optical density. (D) MNV strains were incubated with the indicated concentrations of MAb 5C4 for 30 min at 37°C before a plaque neutralization assay was performed. Virus titers were determined after 48 h of incubation and are shown relative to the absence of antibody. Data are presented as means ± standard errors of the means (SEM) of the results from at least three independent experiments.

To sample the genetic diversity of MNV strains, 10 MNV strains (CR1, CR3, CR6, CR7, MVV1, MNV3, MNV4, S99, WU11, and WU20) were selected for this study based on their capsid sequences (Fig. 2A). Strains clustered into five groups. CR1 was evolutionarily the closest to the reference strain (MNV1; shown in red) and formed group 1. CR7 was evolutionarily farthest from the reference strain and formed group 5. The remaining strains could be grouped into three clusters: group 2 consisted of WU11 and WU20; group 3 included CR6, MNV3, MNV4, and S99; and CR3 was the only member of group 4 (Fig. 2A). Analysis of the VP1 capsid domain sequences confirmed earlier findings (33) that the S domain was highly conserved and that capsid sequence diversity was limited to the P domain. Amino acid differences between strains clustered near the highly flexible E′-F′ loop. Interestingly, only CR7 had the previously identified MNV1 D348E mutation, which mediated neutralization escape for MAbs 4F9 and 2D3 (31) (see Fig. S1 in the supplemental material).

10.1128/mSphere.00334-17.1 FIG S1  P2 domain sequence similarity in MNV. The P2 domain sequences of the 10 MNV strains were compared using the constraint-based cobalt multiple alignment tool from the National Center for Biotechnology. Only amino acid residues with differences in at least one of the strains are shown. Download FIG S1, TIF file, 0.4 MB. Copyright © 2017 Kolawole et al. 2017 Kolawole et al. This content is distributed under the terms of the Creative Commons Attribution 4.0 International license.

Given the high conservation of the NoV S domain (33), we tested the hypothesis that anti-MNV1 S domain antibody 5C4 would bind to a wide range of strains of both murine and human NoVs. To that end, MAb 5C4 was tested for reactivity to 10 MNV and 5 HuNoV strains by enzyme-linked immunosorbent assay (ELISA). Mock lysate was used as a negative control, while MNV1 was used as a positive control. As expected, there was no reactivity for the negative control but binding to MNV1 was observed. MAb 5C4 also cross-reacted with the other nine MNV strains, albeit with slightly differing intensities (Fig. 2B). The cross-reactivity of MAb 5C4 further extended to all HuNoV VLPs tested, namely, the genogroup I Norwalk virus (NV) strain and four genogroup II strains from three genotypes (i.e., Vietnam026 [GII.10], Hiro [GII.12], 2006-Saga [GII.4], and 2012-Sydney [GII.4]) (Fig. 2C). These data provide biological confirmation of the high degree of antigenic conservation of the S domain, which extends across genogroups and genotypes (34). This broad reactivity is consistent with the pan-genogroup reactivity observed previously with MAb TV20, another NoV S domain antibody (35). All MNV strains were next tested against MAb 5C4 in plaque neutralization assays. Similarly to MNV1 (31), none of the other nine MNV strains were neutralized (Fig. 2D). Taken together, these data suggest that MAb 5C4 is a broadly reactive antibody with pan-NoV reactivities that recognized the S domains of MNV and HuNoV strains belonging to different genogroups. This property makes the antibody a valuable reagent for diagnostic applications.

### Monoclonal antibodies 4F9 and 2D3 are broadly neutralizing.

**FIG 3:**
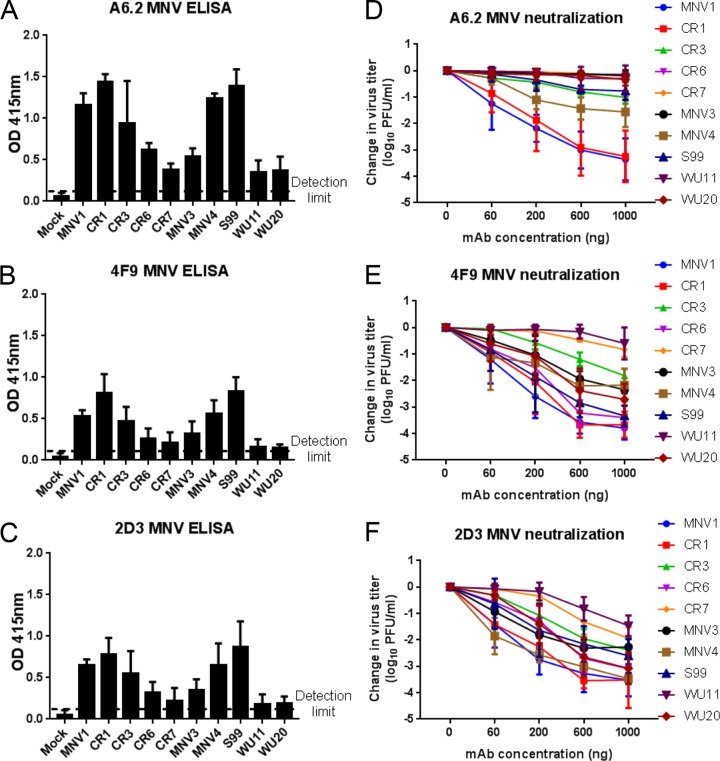
Cross-neutralizing activities of anti-MNV1 MAbs. (A to C) Plates were coated with equal amounts of MNV strains and tested for reactivity to 1 µg per well of MAbs A6.2 (A), 4F9 (B), and 2D3 (C). MNV1 and mock lysate were used as positive and negative controls, respectively. The dashed line represents twice the absorbance values at 415 nm (*A*_415_) for the negative control and indicates the threshold for a positive signal. (D to F) MNV strains were incubated with the indicated concentrations of MAbs A6.2 (D), 4F9 (E), and 2D3 (F) for 30 min at 37°C before a plaque neutralization assay was performed. Virus titers were determined after 48 h of incubation and are shown relative to the absence of antibody. Data are presented as means ± SEM of the results from at least three independent experiments.

To determine the cross-reactivity and neutralizing abilities of anti-MNV1 MAbs A6.2, 2D3, and 4F9 against nine other MNV strains, ELISA and antibody neutralization assays were performed. For the ELISA, MNV1 was again used as a positive control; reaching absorbance readings far above that of mock lysate, the negative control (Fig. 3A to C). For the other MNV strains, antibody reactivity correlated well with the sequence similarity observed in the MNV P2 domain (Fig. S1). CR7, WU11, and WU20 were the least reactive with MAbs A6.2 (Fig. 3A), 4F9 (Fig. 3B), and 2D3 (Fig. 3C). In contrast, the three antibodies were equally reactive with CR1, MNV4, and S99 compared to MNV1, while intermediate levels of binding to CR3, CR6, and MNV3 were observed (Fig. 3A to C). No binding of the three MAbs to HuNoV VLPs was observed (data not shown). These data demonstrate that anti-MNV1 MAbs A6.2, 2D3, and 4F9 bind other MNV strains, albeit with different efficiencies, consistent with the previous classification of these strains into one serogroup (36).

The neutralizing activity of each of MAbs A6.2, 2D3, and 4F9 was next tested against all 10 MNV strains. The antibodies showed a range of neutralization activities in a concentration-dependent manner (Fig. 3D to F). As expected from the very high (95%) sequence identity of the P2 domain, MAb A6.2 neutralized CR1 as effectively as MNV1 (Fig. 3D). Intermediate neutralization was observed for MNV4, CR3, and S99, while CR6, CR7, MNV3, WU11, and WU20 were not neutralized by MAb A6.2 (Fig. 3D). In contrast, MAb 4F9 neutralized 8 of 10 MNV strains, including MNV1, by at least 1 log (Fig. 3E). Only CR7 and WU11 were not neutralized by MAb 4F9 (Fig. 3E). Interestingly, MAb 2D3 broadly neutralized all 10 MNV strains by at least 1 log (Fig. 3F) despite numerous amino acid differences in the P2 domain sequence of these strains (Fig. S1). Taken together, these data demonstrate that MAb 2D3 and, to a lesser extent, MAb 4F9.4, but not MAb A6.2, broadly neutralized MNV strains.

### Modeling of MAb 2D3.

To investigate the atomic details of MAb 2D3 binding and escape from neutralization, an approximate model was made for MAb 2D3 using the previously determined amino acid sequence (31) and the antibody modeling server called PIGS (Prediction of Immunoglobulin Structures) (37). As shown in Fig. S2, the light chain variable domain sequence was found to be nearly identical to that of 2AEQ in the PDB database and the heavy chain variable domain sequence structure was found to be most similar to the structure of 1IBG. In the heavy chain, the CDR1 and CDR2 loops are identical in length. The sequences of the CDR1 loops differ only by a conserved change of a Ser to a Thr. The CDR2 loop contains a less conserved change of a Pro to an Ala. Nevertheless, the model is likely to be highly accurate for much of this domain. As expected, the largest divergence is in the CDR3 loop of the heavy chain. The flanking regions of the loop are well conserved. However, MAb 2D3 contains four additional residues in the loop itself. These four additional residues were added manually to the loop structure, given the realization that they are more representative of place markers for the general composition of the loop rather than a true representation of the structure. Indeed, we previously found that the heavy chain CDR3 loop can move when it binds to the epitope (38, 39). It is interesting that the CDR3 loop contains a relatively high percentage of aromatic and hydrophobic residues (YYDYAVDYW). This is very similar to our previous observation of the heavy chain CDR3 loop in the A6.2 antibody, where we proposed that the CDR3 loop binds in a hydrophobic patch between the A′-B′ and E′-F′ loops (15).

10.1128/mSphere.00334-17.2 FIG S2  2D3 structural modeling performed using the online resource PIG (37). The PDB structure of 2AEQ had nearly complete sequence identity and nearly identical CDR loop lengths. Therefore, it makes for an excellent approximation of the 2D3 structure. The heavy chain variable region was also nearly identical to that of the structure of PDB 1IBG in the CDR1 and CDR2 loops, but 2D3 was four residues longer in the CDR3 loop. Download FIG S2, TIF file, 0.4 MB. Copyright © 2017 Kolawole et al. 2017 Kolawole et al. This content is distributed under the terms of the Creative Commons Attribution 4.0 International license.

### Modeling of the 2D3/MNV complex.

**FIG 4:**
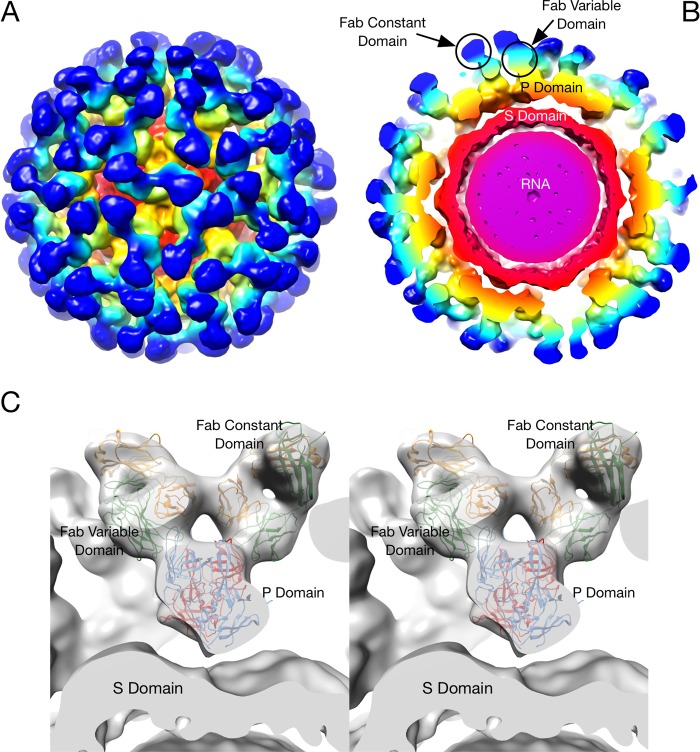
Cryo-EM image reconstruction of the 2D3/MNV complex fitted with the atomic models of the MNV P domain dimer and two of the MAb 2D3 Fabs. (A) Surface rendering of the entire virus colored according to the particle radius. (B) Center section of the same structure. The Fab constant domains are shown in dark blue, the variable domain in cyan, and the P domain in yellow/orange. The shell domain is orange, and the RNA interior is purple. (C) A stereo image of the Situs fitted components corresponding to the cryo-EM density data. The heavy chains are shown in green, the light chains in yellow, and the two conformations of the P domain, A and B, in red and blue, respectively.

The cryo-EM image reconstruction of the 2D3 Fab/MNV1 complex (Fig. 4) shows a number of similarities to and differences from our previous MAb A6.2/MNV structure (25, 40). As we previously observed with MNV1 (25, 40), rabbit hemorrhagic disease virus (40), and GII.10 Vietnam026 HuNoV VLP (41), the P domain was found to be floating above the shell domain by ~16 Å (Fig. 4B and C). This flexibility and the inherent flexibility within the Fab lead to lower resolution in the Fab fragment than in the more rigid shell domain. Also, similarly to the A6.2 Fab/MNV1 complex, the antibody binds to the very top of the P domain. The molecular envelope of the Fab is well defined, with even the expected hole in the flexible elbow region present. Using Situs (42) to fit the variable domain separately from the constant domain, the apparent elbow angle was found to be 140°, well within the expected range of 180° to 130° (43).

**FIG 5:**
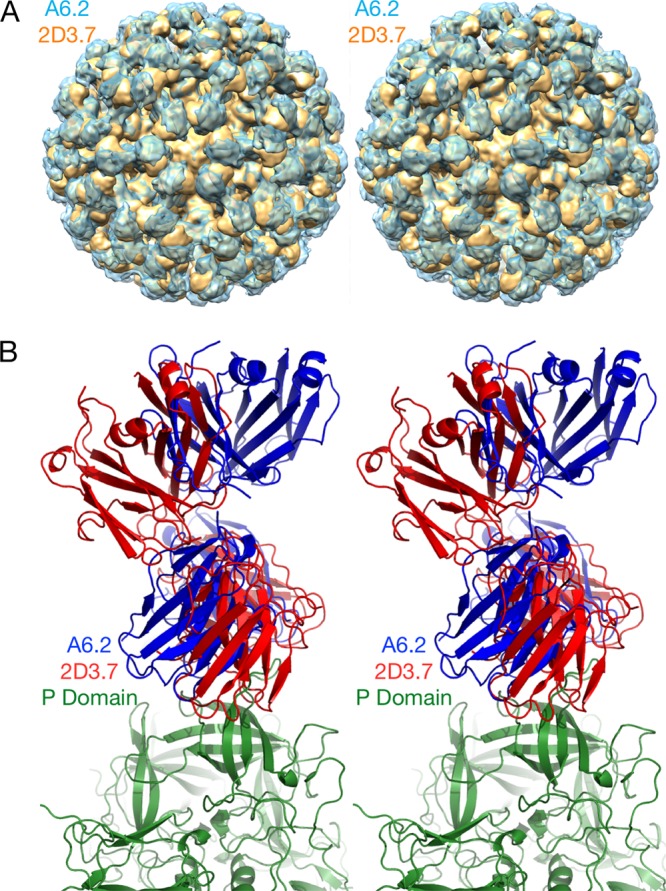
Comparison of the binding conformations of MAbs A6.2 and 2D3. (A) Stereo image of surface rendering of the two MNV complexes, with MAb 2D3 shown in yellow and MAb A6.2 in transparent blue. (B) Stereo image of the Situs fitted models with MAb A6.2 shown in blue and MAb 2D3 in red. The P domain is shown in green.

As shown in Fig. 5, the binding location of MAb 2D3 was very similar to that of MAb A6.2 in spite of the fact that the escape mutants corresponding to the one antibody do not affect the neutralization of the other (31). MAb A6.2 binds in a more vertical orientation at the very tip of the P domain, while MAb 2D3 binds at more of an angle and contacts more of the side of the A′-B′/E′-F′ loops.

**FIG 6:**
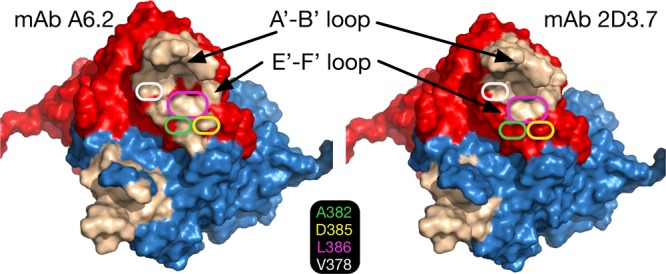
Comparison of the interactions between MAb 2D3 and the open and closed P domain conformations of MNV. The model shown represents the results of application of the molecular dynamics flexible fitting method (MDFF) (44) to the starting Situs model. The light and heavy chain variable domains of MAb 2D3 are shown in orange and green, respectively. The open and closed conformations of the P domain are shown in red and blue, respectively. Also noted are the locations for MAb 2D3 escape mutations V339I and D348E (shown with mauve and yellow spheres, respectively). Note that the CDR3 of the Fab loop fits well between the A′-B′ and E′-F′ loops of the open conformation of the P domain but overlaps with the A′-B′ loop in the closed conformation.

To more accurately define the contact area of Fab 2D3 on the P domain, the PISA assembled model was refined using the molecular dynamics flexible fitting (MDFF) method (44). The MDFF method allows for an all-atom energetically relaxed conformational contact interface while being restrained by the cryo-EM density. This restrained flexibility is important, since, as we observed in the case of Fab17 bound to human rhinovirus 14 (HRV14) (39, 45), even if both the antigen and antibody structures are known, there is some minor conformational rearrangement, in both the Fab and the P domain, to optimize the interaction. As shown in Fig. 6, the contacts between Fab and the P domain in the open conformation are far superior to those in the closed conformation. In the open conformation, the Fab CDR3 loop fits between the A′-B′ and E′-F′ loops. In contrast, the E′-F′ loop in the closed conformation is vertical and clashes with the CDR3 loop even after dynamic simulation and energy minimization. While it is expected that the conformation of the CDR3 loop would change upon antigen binding, there is simply not enough space to accommodate the CDR3 loop and the E′-F′ loops in this configuration. For this reason, similarly to what we found with MAb A6.2 (31), it seems more likely that the antibody is binding to the open conformation where the A′-B′ and E′-F′ loops are splayed. Further, as noted above, the CDR3 loop of MAb 2D3 contains a high density of aromatic and hydrophobic residues. As was also the case with MAb A6.2 (31), this orientation places the hydrophobic CDR3 loop into the hydrophobic cleft between the A′-B′ and E′-F′ loops.

**FIG 7:**
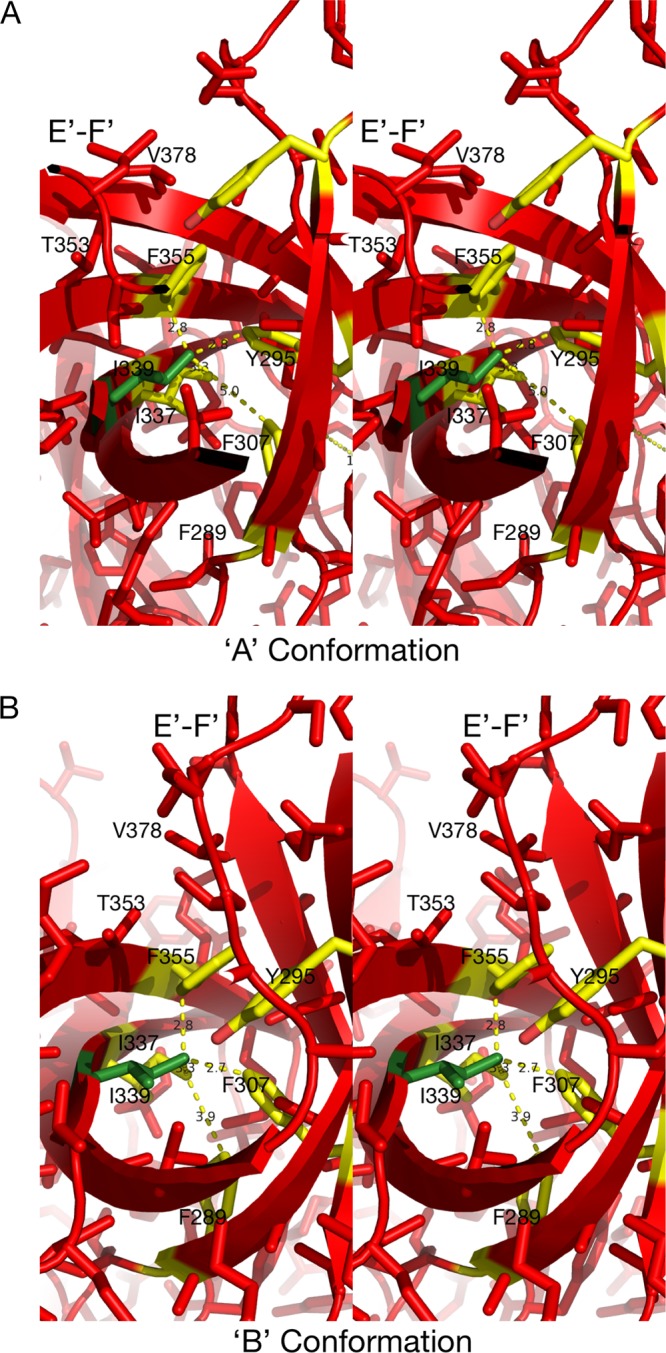
Comparison of the MAb A6.2 and MAb 2D3 antibody contact areas. To calculate the contact areas, the refined Fab/MNV models were analyzed using PDBePISA (46) for just the Fab contacts with the open conformation. The surface areas of the open and closed conformations are shown in red and blue, respectively. The residues in the open conformation that contact the bound antibodies are shown in wheat color. Also noted are the locations for the MAb A6.2 escape mutations on the E′-F′ loop.

To better highlight the epitope differences between the A6.2 (31) and 2D3 MAbs, contact residues were mapped out on the surface of the P domains (Fig. 7). Contact residues between each Fab and the open conformation were determined using PDBePISA (46) (http://www.ebi.ac.uk/pdbe/pisa/) and are denoted on both the open and closed conformations as the wheat-colored surface in Fig. 7. Both Fabs made significant contact with the A′-B′ loop and some contacts around the depression between the A′-B′ and E′-F′ loops. However, 2D3 appears to bind further down in this depression, whereas A6.2 binds more to the outer rim.

What is particularly interesting is that the escape mutations corresponding to A6.2 do not affect 2D3. In the case of A6.2, there are four escape mutation sites on the E′-F′ loop: A382, D385, L386, and V378. In the model of A6.2 binding to the open conformation, V378 lies between the A′-B′ and E′-F′ loops and the hydrophobic side chain points toward F355. With both A6.2 (31) and 2D3 antibodies, the backbone atoms make contact with the antibody but the side chain is mostly buried. We had previously proposed that the V378F mutation may be affecting the opened/closed conformational transition rather than just sterically blocking antibody binding (15). Similarly, we proposed that the A382K/R escape mutation may not be directly affecting binding but, again, forces the P domain into the closed conformation that is not favored by the antibody. A382 is adjacent to the two antibodies but not in contact with either antibody. The major difference between the two antibodies is that A6.2 contacts D385 (escape D385E) whereas 2D.3 does not. Finally, L386F (escape L386F) lies between the A′-B′ and E′-F′ loops and is in contact with both antibodies, mainly via the backbone atoms. It appears that while a number of these A6.2 escape mutant residues contact 2D3, their presence is apparently not critical for binding. At minimum, these results suggest that antibodies can have disparate critical binding residues in spite of having nearly identical contact regions.

### MNV allostery-like escape mutations block MAb 2D3 binding.

**FIG 8:**
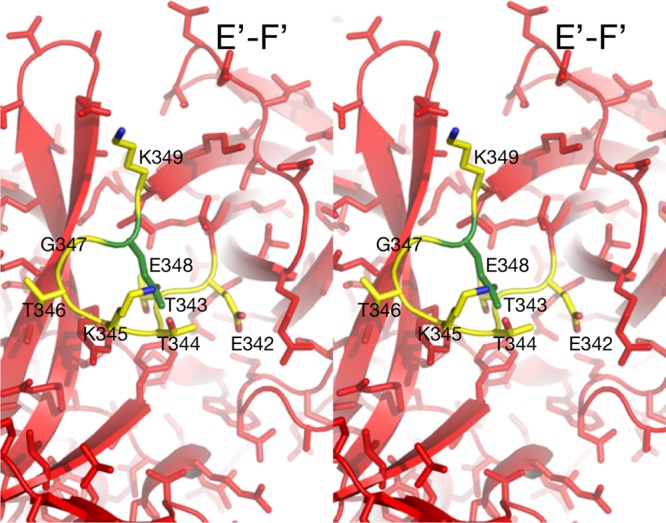
Stereo images of the environments around the MAb 2D3 escape mutants in the two P domain conformations. (Left) The V339I mutation in the context of the open conformation, with the modeled mutation shown in green and the possible residues affected by the mutation highlighted in yellow. (Right) In contrast to the open conformation, there is a significantly more space around I337 in the closed conformation that could accommodate the additional methyl group.

To understand the mechanism of escape from 2D3 neutralization, the modeled structure of the P domain containing escape mutants V339I and D348E with the Fab was examined. In contrast to our results obtained with MAb A6.2 (31), neither of the escape mutations corresponding to MAb 2D3 is in contact with the bound antibody (Fig. 6 to 8). Both V339 and D348 lie beneath the E′-F′ loop and therefore are impacting the antigenic site in an allostery-like manner by changing longer-range mechanical couplings. In the case of the V339I mutation, it appears that the open conformation is less tolerant of the bulkier Ile residue than the closed conformation (Fig. 8). In the open conformation, residues such as F355 have to move away from I339. However, in their positions immediately above I339, residues T353 and V378 would limit such movement. In contrast, the E′-F′ loop in the closed conformation is lifted off the rest of the P domain, freeing space around F355 to allow movement away from I339. From these results, it appears that the V339I mutation would cause the P domain to favor the closed conformation over the open conformation. Thus, it is possible that this escape mutation would make it harder for MAb 2D3 to bind to its favored open conformation where it has access to the hydrophobic patch between the A′-B′ and E′-F′ loops.

**FIG 9:**
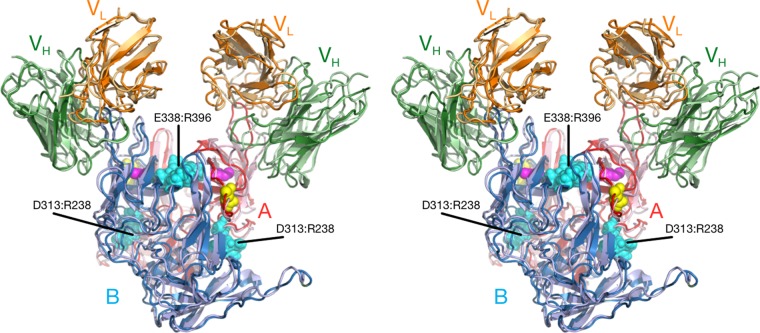
Stereo image of the local environment of the D348E mutation. The modeled mutation is shown in green, and possible contacts are highlighted in yellow. This mutation lies on a loop that is on the side of the P domain and not near the bound 2D3 antibody.

With the D348E escape mutation, it is more difficult to see a clear path between the effects of the mutation and MAb 2D3 binding (Fig. 9). D348 lies on an exterior loop beneath the E′-F′ loop. In Fig. 9, the rotomer for the side chain that best fits into the structure is shown. What is clear from this model is that the larger side chain would more than likely disrupt the conformation of that loop. It is possible that, to accommodate the larger side chain, the backbone of the downstream β-strand is disrupted such that it affects the interactions with the E′-F′ loop in the open conformation (the conformation shown in Fig. 9). Because this mutation is so distal to the bound antibody, MD studies focused on I339. Far-more-extensive MD studies of D348 are under way. Taken together, these data suggest that both escape mutants likely cause changes in the flexible P domain loops.

### Dynamic simulations of the effects of the V339I escape mutation.

**FIG 10:**
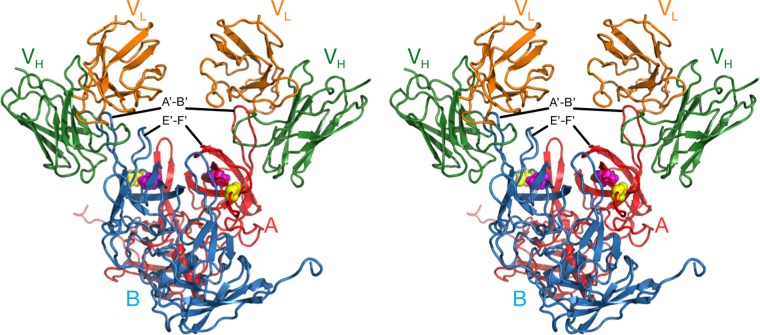
Stereo image of the effects of the V339I escape mutation on the *in silico* molecular dynamics of the 2D3 Fab/MNV complex. The variable regions of the heavy and light chains are shown in green and orange, respectively, and the open and closed conformations in red and blue, respectively. The minimized wild-type structure is shown in the darker hues, while the structures seen after the molecular dynamics experiments are shown in lighter hues. While the minimized wild-type structure remained relatively stable (RMS deviation of ~1 Å), the V339I structure showed significant long-range disruptions (RMS deviation of ~2.5 Å). The salt bridges affected by the mutation are shown as cyan spheres labeled with the names of the amino acid pairs. The V339I and D348E escape mutations are denoted by mauve and yellow spheres, respectively.

Since the V339I escape mutation is not in contact with the 2D3 MAb, it is highly likely that the V339I mutation affects binding via long-range effects on the P domain structure. To investigate this possibility, molecular dynamics flexible fitting was applied to the 2D3 Fab/MNV1 complex. When molecular dynamic simulations were run for 20 ns, after 10,000 steps of minimization and 1.1 ns of equilibration performed on the wild-type (wt) complex, the structure was stable, with a root mean square deviation (RMSD) of ~1 Å. However, when the V339I mutation was placed in the wild-type structure, minimized, and then subjected to the same molecular dynamics protocol, the structure was far less stable, with an RMSD of ~2.5 Å. As shown in Fig. 10, the structural changes were not limited to the area immediately around the V339I mutation but were rather seen throughout the P domain structure and up into the Fab contact region. As discussed above, V339 is in a fairly confined area such that the presence of a larger side chain would be expected to be disruptive. However, it is surprising to see the extent of the changes caused by this relatively conserved V339I mutation.

The final fitted average structure from the MDFF simulations does not change the asymmetric binding observed with the cryo-EM reconstruction. The results reflect an anchoring of the P domain’s B chain (closed conformation) to Fab via the intercalation of the Fab CDR3 loop between the A′-B′ and E′-F′ loops, and a relaxation of the open conformation (chain A) is assisted by a loss of the buried salt bridge network at the interface. Only one salt bridge was conserved, A:Arg386 and B:Glu338, at a shortened length of 2.98 Å. A new salt bridge on the exposed surface between A:Arg238 and B:ASP313 was identified. The symmetric, A:Glu338 and B:Arg386 interactions were lost in this final conformation as the A and B chains relaxed and the orientations and distances of the A′-B′ and E′-F′ loops of each chain separated relative to the other. The presence of this complex implies that antibody binding to the MNV dimer requires it to “unzip” its interfacial salt bridge network and to shorten the conserved A:Arg396-B:Glu338 interaction, thereby giving the A′-B′ and E′-F′ loops the flexibility needed to adjust to the intercalation of the CDR3 loop. To further investigate the importance of the salt bridge network and the conformational sensitivity of the MNV P domain, MDFF calculations were also performed for the Fab2D3/MNV1 complex with the V339I mutation and with the closed conformation of the P domain.

To make the V339I mutation, the Mutator Plugin in VMD (47) was used to create a starting structure from the Fab2D3/MNV1 cryo-EM reconstruction. A short (5,000-step) minimization procedure was performed using the Generalized Born Implicit solvent in NAMD (48) to relax the new side chain before the molecular dynamics flexible fit method was applied to the cryo-EM map. The MDFF procedure used was the same as that used for the wild type. For the V339I mutation, the RMSD of the backbone atoms was 2.5 Å, a value larger than that observed for the wild type. The final average structure was obtained from the last 2 ns of the simulations.

The final conformation of the Fab2D3/MNV1 complex with the V339I mutation retained the asymmetric starting association but showed less relaxation of the P domain interface. The conserved salt bridge between A:ARG396 and B:GLU338 was present as well as one interaction between A:GLU338 and B:ARG396. The larger side chain of the mutation on chain A limits the flexibility of the P domain interface and hinders the “unzipping” of the salt bridge network, thereby forcing the interface to retain more of its dimer characteristics.

In the final study procedure, the P domain of the Fab2D3/MNV1 complex from the reconstruction was replaced by the closed conformation of MNV1 from the crystal structure (PDB identifier [ID] 3lqe). The MDFF simulation protocol used was the same as that used as described above for the wild-type and the V339I simulations. The salt bridge network at the interface of the P domain of the final average structure contains more interactions than were found for the starting structure with the open conformation of the P domain but fewer than those that were found for the system with the V339I mutation (Table 1).

**TABLE 1:** Distances of the salt bridge interactions at the chain A-chain B interface of P domain of Fab2D3/MNV1 final conformations from the MDFF simulations^a^

Chain A; open conformation	Chain B; closed conformation	Cryo-EM reconstruction R (Å)	Open conformation R (Å)	Closed conformation fitted R (Å)	Closed conformation V339I mutation R (Å)
ASP313 [OD1]	ARG 238 [NH1]			3.57	
ASP313 [OD2]	ARG 238 [NE]	3.14			
ASP313 [OD2]	ARG 238 [NH1]	3.28			
ASP313 [OD2]	ARG 238 [NH2]	3.93			
GLU338 [OE1]	ARG396 [NE]			3.90	
GLU338 [OE1]	ARG396 [NH1]	2.76			
GLU338 [OE1]	ARG396 [NH2]	3.65		3.40	
GLU338 [OE2]	ARG396 [NH1]	2.63			
GLU338 [OE2]	ARG396 [NH2]	3.12			3.44
GLU338 [OE2]	ARG 437 [NH2]	2.75			
ARG396 [NH1]	GLU338 [OE1]	2.85			3.92
ARG396 [NH1]	GLU338 [OE2]	3.16		3.91	3.31
ARG396 [NH2]	GLU338 [OE2]	2.58		3.57	3.57
ARG396 [NH2]	GLU338 [OE1]	3.81	2.98		
ARG396 [NE]	GLU338 [OE2]			3.49	

a The structures are identified by the conformation of the P domain, A, B, and V339I mutation. Empty cells indicate the lack of interactions.

## DISCUSSION

There has been growing interest in developing broadly neutralizing monoclonal antibodies for use in antiviral therapeutics. Such antibodies for HuNoV would be of high impact since no vaccines against HuNoV have yet been approved for use (49) and new NoV strains emerge every 2 to 4 years (16). Thus, a potent, broadly neutralizing anti-HuNoV MAb would be of great value to limit HuNoV infections in highly vulnerable populations (50). Theoretically, such a MAb should target a highly conserved region of the virus capsid or create a bottleneck for virus escape.

To evaluate this hypothesis, we tested a panel of MAbs for their reactivity and neutralization capacity against 10 different MNV strains and five HuNoV VLPs representing multiple HuNoV genotypes and strains. MAb 5C4 recognized a shared epitope of NoV genogroups GI, GII, and GV since it cross-reacted with all the MNV and HuNoV strains tested. This functionally supports reports that the NoV S domain is highly conserved genetically (33, 51). However, MAb 5C4 was nonneutralizing and thus unable to limit NoV infections. Although MAb 5C4 and antibodies with similar features do not have therapeutic potential, they are being developed as useful tools for NoV diagnostics (52, 53).

While MAb A6.2 was able to bind to all MNV strains tested, it was able to effectively neutralize only MNV1, which it was raised against, and the very closely related CR1 strain. In addition, MNV1 was able to escape MAb A6.2 neutralization in just three passages (15, 31). MAb A6.2 binds to the tip of the highly flexible A′-B′ and E′-F′ loops of the P domain, and all of the known escape mutants corresponding to MAb A6.2, residues 378, 382, 385, and 386, are located in the E′-F′ loop (15, 30, 54). The only residue shared between MNV-1 and CR1, but not with any of the other nonneutralized MNV strains, was V378. Neutralization and binding are roughly correlated (Fig. 3), and therefore V378 seems to be important for A6.2 binding.

In contrast, MAb 2D3 has features consistent with therapeutic use. MNV1 had far more difficulty in overcoming neutralization, as it took over 20 passages for MNV1 to escape MAb 2D3 neutralization (15, 31). While MAb 2D3 contacts the E′-F′ loop of the P domain similarly to MAb A6.2, MAb 2D3 appears to bind deeper in the crevice between the A′-B′ and the E′-F′ loops (Fig. 7). Since none of the escape mutations corresponding to MAb A6.2 affect MAb 2D3 neutralization (31), those contact residues in common between the two antibodies apparently do not contribute significantly to 2D3 binding. CR7 has the highest number of amino acid differences compared to MNV1 in the P2 domain and therefore is among those least affected by MAb 2D3. This correlation between sequence divergence and MAb 2D3 resistance could be due to an additive effect of these changes on the conformation of the P domain or to antibody binding or both. Interestingly, CR7 is the only MNV strain that has Glu instead of Asp at position 348. Since D348E is one of the only two reported escape mutants corresponding to MAb 2D3 (31), it may explain why MAb 2D3 was only weakly able to neutralize CR7.

MAbs A6.2 and 2D3 demonstrate how antibodies can bind to similar surfaces of the epitope and yet have very different residues critical for binding. While both antibodies contact the A′-B′ loop, none of the escape mutations lie on this loop. Changes in 297 were lethal when residues 297 to 300 were mutated (15), while the remaining residues did not affect MAb A6.2 binding. Among the four MAb A6.2 escape mutation sites (A382, D385, V378, and L386), only 2D3 fails to contact A382 and V378. Therefore, the two antibodies appear to be binding to the same site of the P domain but the critical binding determinants are very narrow and nonoverlapping. This is in stark contrast to results seen with other viruses, such as human rhinovirus 14, where clusters of monoclonal antibodies corresponding to the same epitope were found to have the same key binding residues (55, 56). One reason for this difference might be the fact that the antigen binding surfaces on HRV14 are far less flexible than the MNV P domain. In this way, HRV14 presents essentially one conformation to the immune system whereas MNV may present several conformations, resulting in antibodies with different critical binding residues.

The 2D3/MNV complex and the MAb 2D3 neutralization escape mutations pose perplexing questions. Why was the virus unable to mutate any of the 2D3 surface contact residues to block antibody binding? How do these distant and buried mutations block antibody binding? One possible reason for such a restricted escape mutation repertoire is that the receptor binds to the same surface as MAb 2D3 or to overlapping surfaces. It may be that the residues critical for 2D3 binding (but not for MAb A6.2 binding) are the same as those used by the receptor. It would follow that the escape mutations corresponding to 2D3 conformationally disrupt the epitope structure sufficiently to block antibody binding but not sufficiently to affect the receptor binding. While recombinant viruses retained the V339I, D348E, A382K, and L386F mutations over 10 passages in the absence of antibody (data not shown), there may be more subtle effects on viral fitness that are revealed only after longer passaging or in different contexts. Proof that 2D3 binds to the receptor contact region is beyond the scope of this current study and will require elucidation of the structure of the virus/receptor complex.

It is also conceivable that the MAb 2D3 escape mutations V339I and D348E affect the conformational equilibrium between the open and closed conformations. As an example of how this might work, perhaps the receptor prefers the closed conformation and the escape mutations shift the structural equilibrium toward the closed conformation and thereby block MAb 2D3 binding while having minimal effect on receptor binding. In this way, perhaps escape mutations are not found between the A′-B′/E′-F′ loops, where most of the antibody contacts lie, because they have a deleterious effect on the closed conformation that the receptor requires. Nevertheless, it is extremely unusual that none of the surface contact residues corresponding to 2D3 were found to mutate to block antibody binding and that the virus had to resort to a less direct, allostery-like escape mechanism. The analogy of this escape mechanism to allostery, even though it is not due to an effector binding away from the “active” site, is a handy way to describe mutations that induce long-distance communications via conformational changes (see, e.g., reference 72).

The results from the three flexible fit molecular dynamics simulations, performed using the cryo-EM map as a constraint, indicate that the Fab2D3/MNV1 complex is altered by the flexibility of P domain dimer interfacial interactions, in particular, that of the salt bridge network that undergoes rearrangements necessary to accommodate the antibody binding. As seen from the P domain open and closed conformations of the crystal structures, the buried dimer interface salt bridge network is complex and dynamic, thus allowing the dimer to adopt more than one conformation. In the case of the V339I mutation, a mutation outside the A′-B′ or E′-F′ antibody binding loops, the salt bridge network restricts the molecular liberation of the interfacial region and may limit the P domain conformational flexibility required for binding.

Side chain interactions in folding configurations differ from those found at interfaces where associations accommodate minor conformational adjustments, usually in side chains, not in the folded state of the protein units. The salt bridge and hydrogen bonding networks at these buried interfaces, devoid of mediating solvent molecules, are specific and complementary. Salt bridges are able to contribute to conformational specificity and to molecular recognition and catalysis. Thus, as discussed by DeGrado et al. (57), salt bridges are difficult to predict because of the cost of formation, dehydration of a basic residue and carboxylate, and the electrostatic and hydrogen bonding interactions, but they may be viable designable interactions.

In summary, there are several key and unique findings reported here. First, it is uncommon to have an exposed antigenic surface that is conserved enough to elicit cross-reactive antibodies. Second, this antigenic site is also highly unusual in that the only escape mutants corresponding to MAb 2D3 found to date, D348E and V339I, arise very slowly in the presence of antibody and are distal to any antibody contact residues. None of the antibody binding contacts mapped out by the cryo-EM structure appear in the escape mutation repertoire. The simplest explanation for this is that residues critical for MAb 2D3 binding largely overlap those necessary for receptor binding and that the effects of mutating those contact residues are lethal. If true, this would be a striking example of a virus with an exposed, conserved receptor binding region(s) that is fully visible with respect to immune recognition. Other examples of this receptor/antibody binding overlap include human rhinovirus 14 (HRV14), where antibodies penetrate the receptor binding canyon (39, 58), and foot-and-mouth disease virus, where antibodies bind directly to the integrin RGD binding motif (Acharya, 1989, no. 4226; Domingo, 1999, no. 4920). Third, the results of *in silico* analysis of the escape mutant suggest that the P domain is more flexible than previously thought and that the dimer interface may play a role in this plasticity. This flexibility might be important not only for antibody binding but for receptor interactions as well. Taken together, the results presented here may be important for NoV vaccine development as well as for understanding virus-receptor interactions.

## MATERIALS AND METHODS

### Cell culture.

Murine macrophage RAW 264.7 cells were purchased from the American Type Culture Collection (ATCC) and maintained as described previously (20, 30). Anti-MNV MAbs A6.2, 2D3, and 4F9 were generated as previously described (20, 31).

### Viruses.

Ten MNV strains were used in this study. MNV1.CW3 (GV/MNV1/2002/USA) (referred to here as MNV1) (18), CR1 (GV/CR1/2005/USA), CR3 (GV/CR3/2005/USA), CR6 (GV/CR6/2005/USA), CR7 (GV/CR7/2005/USA) (36), MNV3, MNV4 (59), S99 (Berlin/06/06/DE) (60), WU11 (GV/WU11/2005/USA), and WU20 (GV/WU20/2005/USA) (36) were amplified in RAW 264.7 cells and used at passage 6 (P6). Concentrated virus stocks were generated as previously described (61). Generation and characterization of recombinant viruses V339I, D384E, A382K, and L386F and of the wt virus were described previously (15, 31). Viruses were passaged 10 times in RAW cells without antibody as previously described (31).

### Phylogenetic analysis.

The capsid protein sequences and P2 domain sequences of the 10 MNV strains were compared using the constraint-based cobalt multiple alignment tool on the National Center for Biotechnology website. The phylogenetic tree of the 10 MNV strains was constructed based on the capsid sequences using the “phylogenetic tree” function within the cobalt suite.

### Human norovirus virus-like particles (VLPs).

Five HuNoV VLPs used in the study were graciously provided by Grant Hansman from the University of Heidelberg, Germany. The genogroup I strain Norwalk virus (NV) (GenBank accession number AY502016) and genogroup II strains GII.4 Saga (GenBank accession number BAG70515), GII.4 Sydney (GenBank accession number JX459908.1), GII.10 Vietnam026 (GenBank accession number AF504671), and GII.12 Hiro (GenBank accession number AB044366) VLPs were expressed and purified as previously described (62).

### Plaque assay with neutralization.

Each of the 10 MNV strains (5 × 10^4^ PFU) was subjected to 0, 60, 200, 600, or 1,000 ng of purified MAbs 5C4, A6.2, 2D3, and 4F9 followed by enumeration by plaque assay as previously described (63).

### ELISA.

The enzyme-linked immunosorbent assay (ELISA) was conducted as described previously (20) with the following modifications. Each well of a 96-well enzyme immunoassay/radioimmunoassay (EIA/RIA) plate was coated with 2 µl of concentrated virus of each MNV strain or 1 µg of each HuNoV VLP diluted into 100 μl phosphate-buffered saline (PBS) and incubated for 24 h at 4°C.

### MNV purification.

Virus stocks of MNV1 were propagated by infecting RAW 264.7 cells grown in suspension culture in spinner flasks using suspension in modified minimal essential medium (MEM) (United States BioLogicals, Swampscott, MA) supplemented with 10% bovine serum (Life Technologies Corporation, Carlsbad, CA), 100 U penicillin/ml, and 100 µg/ml streptomycin. Approximately 3 liters of RAW 264.7 cells at ~10^6^ cells/ml was harvested by centrifugation at 600 × *g* for 10 min. The cells were washed with PBS and infected with MNV1 at a multiplicity of infection (MOI) of 5. The infected cells were incubated at room temperature for 1 h before being supplemented with 100 ml of MEM (Life Technologies Corporation, Carlsbad, CA) buffered with 25 mM HEPES. The infected cells were incubated at 35°C for 10 to 12 h in a shaking water bath and were then frozen by storage at −80°C. Virus particles were released by the use of three freeze-thaw cycles. The virus lysate was then centrifuged at 10,000 rpm to remove cellular debris. The supernatant was then treated with DNase I (10µg/ml) and MgCl_2_ (5 mM) and allowed to incubate at room temperature for 1 h. After digestion, 10 mM EDTA and 1% lauryl sarcosine (final concentrations) were added to the supernatant. This solution was then centrifuged at 48,000 rpm using a 70Ti rotor (Beckman Coulter, Inc.) for 2.5 h. The resulting pellet was resuspended in PBS (~250 to 300 µl), allowed to rest at 4°C for several hours, applied to a continuous sucrose gradient (7.5% to 45%), and centrifuged at 36,000 rpm for 2 h in an SW41 Ti swinging-bucket rotor (Beckman Coulter). The virus band was then collected with an Isco gradient fractionator, and the virus-containing fractions were pooled, diluted with PBS, pelleted at 48,000 rpm for 2 h, and resuspended in 200 µl PBS. Each 3-liter volume of cell culture yielded 0.5 to 1.0 mg of purified virus with a particle/PFU ratio of less than 100. Note that the ultracentrifugation step, compared to concentration procedures performed via the use of Centricon ultrafiltration units (Millipore Corp., Billerica, MA), had no effect on viral infectivity.

### Purification of MAbs.

IgG MAbs 5C4 and A6.2 were grown and purified as previously described (31).

### Production and purification of MAb 2D3.

Since MAb 2D3 is an IgA antibody, it could not be purified using protein A/G affinity chromatography. Antibodies were grown in Bioreactor CELLine CL 1000 flasks (Sigma-Aldrich) as described previously. IgA MAb 2D3 was first precipitated from the hybridoma cell culture supernatant with a 50% (final concentration) saturated solution of ammonium sulfate. The precipitate was collected by centrifugation and dialyzed against PBS. 2D3 was then purified using an S-200 size exclusion column (5-cm diameter by 50-cm height) equilibrated with PBS. Protein elution was monitored via *A*_280_ analysis, and fractions containing IgA were identified by SDS-PAGE (with or without a reducing agent). Purified IgA antibody solution was dialyzed against 10 mM Tris (pH 7.5). Immediately before digestion, 1 M stock sodium citrate (pH 4.0) was added to yield a final concentration of 0.1 M. This limited the exposure of the antibody to this low-pH condition. The antibody was then digested with pepsin (pepsin/Ab ratio, 1:25 [wt/wt]) for 17 h at 37°C. Digestion was stopped by neutralization of the solution with 1 M Tris buffer (pH 9.0). The digested IgA solution was then dialyzed against 50 mM Tris-HCl (pH 7.6) and applied to a Mono-Q column equilibrated with the same buffer. Bound IgA Fab was eluted with a NaCl gradient in the same Tris buffer. The main species after digestion is an (Fab′)_2_ that is held together via heavy chain disulfide bonds. Therefore, the antibody was treated with 25 mM β-mercaptoethanol prior to addition to MNV.

### Cryoelectron microscopy and image reconstruction.

Data were collected at the W. M. Keck Center for Virus Imaging at the University of Texas Medical Branch at Galveston (UTMB). Small (~2.5-to-3.5-ml) aliquots of ~5 mg/ml solutions of purified, infectious MNV samples were adhered to holey carbon-coated electron microscope grids and vitrified in liquid ethane.

MNV was vitrified using a Vitrobot Mark IV unit as reported previously (64) on 1.2/1.3 C-flat grids (Protochips, Raleigh, NC). The humidity in the Vitrobot chamber was set to 100%, and the temperature was 22°C. Frozen grids were stored under liquid nitrogen and transferred to a cryo-specimen 626 holder (Gatan, Inc., Pleasanton, CA) before they were loaded into a JEM-2200FS electron microscope, equipped with an in-column energy filter (omega type) and a field emission gun (FEG), operating at 200 keV. Grids were maintained at near-liquid-nitrogen temperature (−172 to −180°C) during imaging. Images were acquired at a nominal microscope magnification of ×40,000 using a DE-20 camera system (Direct Electron, LP, San Diego, CA) with approximately 32 electrons/Å^2^ total exposure; the pixel size corresponded to 1.49 Å on the specimen scale. An in-column omega electron energy filter was used during imaging with a zero-loss electron energy peak selected using a 20-eV slit. Images were collected with defocus values ranging from 0.7 to 2.0 µm.

Images were acquired using movie mode (continuous streaming at 25 frames per s delivering in-line dose fractionation); individual frames from each image were aligned and adjusted to account for radiation damage with a DE_process_frames.py script developed by Direct Electron. In the processed images, visible Thon rings extended to better than 6 Å.

Images of MNV were boxed out from 300 micrographs. Image processing and image reconstruction were performed using EMAN2 (65). The final data set contained 2,333 virions extracted with a box size corresponding to 2.5× the particle diameter. Resolutions were assessed by calculating the Fourier shell correlations (FSC) of the three-dimensional (3D) reconstructions and applying cutoff values of 0.5 and 0.143 (see Fig. S3 in the supplemental material). This yielded calculated resolutions of 9.9 Å and 8.7 Å, respectively. The 3D maps were surface rendered and displayed with a threshold of a standard deviation of 1 (1σ) in CHIMERA (66), which accounted for ~100% particle volume. The map has been deposited in the PDB database with accession number EMD-8842.

10.1128/mSphere.00334-17.3 FIG S3  Fourier shell correlation of the cryo-EM image reconstruction versus resolution. Noted are the estimated resolutions based on 0.5 and 0.143 correlations of 9.9 Å and 8.7 Å, respectively. Download FIG S3, TIF file, 0.1 MB. Copyright © 2017 Kolawole et al. 2017 Kolawole et al. This content is distributed under the terms of the Creative Commons Attribution 4.0 International license.

### Modeling of the 2D3/MNV complex.

The atomic structure of the IgA 2D3 has not yet been determined because of the known difficulty in obtaining homogeneous pepsin cleavage product (67). Therefore, to characterize at least the chemical nature of the 2D3 paratope, the structure of 2D3 was modeled using the online application PIGS (Prediction of Immunoglobulin Structures; http://circe.med.uniroma1.it/pigs/) (37). PIGS found two highly homologous structures in the database that correlated well with the sequence of 2D3: 2AEQ for the light chain and 1IBG for the heavy chain. As shown in Fig. S2, all three of the CDR loops in the light chain and the CDR1 and CDR2 loops in the heavy chain are nearly identical. The only significant deviation is, as expected, seen with the CDR3 loop of the heavy chain. The CDR3 loop of 2D3 is four residues longer, but the residues flanking this insertion are highly conserved. PIG did not build this CDR3 loop, but only modest modification was required to build in the four residues with acceptable geometry using the program COOT (68). The details of this loop are not as important as its general character. Most notably, CDR3 has an unusually high density of aromatics and hydrophobic residues (YYDYAVDYW).

Using this crude model, the Situs package (42) was then used to model the Fab into the corresponding electron density of the 2D3/MNV complex. The variable domain from PIG was combined with the constant domains from our previous A6.2 MNV antibody to create a Fab with the same elbow angle as A6.2. The resulting Fab was then roughly fitted into the molecular envelope, and the variable domain, constant domain, and P domain dimers were allowed to refine independently. The elbow angle of the resulting model was then examined using the program Phenix (69) to see whether the refined domain orientations were within the expected ranges. However, the elbow angle of this model was calculated to be 208°, while nearly all Fabs have an elbow angle between 130° and 180°. Therefore, the original Fab model was then rotated by 180° and refitted into the electron density and Situs refinement was run again on the individual domains. The resulting elbow angle was 140° and is well within the expected range.

### Molecular dynamics flexible fitting of the FabD/MNV complex.

The molecular dynamics flexible fitting (MDFF) method (44) was used to fit the atomic structure to the cryo-EM map. The starting coordinates from the cryo-EM reconstruction described above were used as the starting conformation for the flexible molecular dynamics fit. For this starting structure, the A (open conformation) and B (closed conformation) chains of the P domain are in the open conformation as presented in the previously released high-resolution X-ray crystal structure, PDB ID 3lqe (30). In this starting conformation, FabD’s CDR3 loop is intercalated between the A′-B′ and E′-F′ loops of chain B only; the Fab-MNV interaction is not symmetric with respect to each of the dimeric units. Taube et al. observed a second closed dimer conformation of the P domain of MNV, PDB ID 3lq6, which differs in the conformation of the A′-B′ and E′-F′ loops as shown in Fig. 6 and in the salt bridge network at the interface of chains A and B as illustrated in Table 2. The salt bridges at the interface are buried and “networked” but more complex for conformation A.

**TABLE 2:** Distances of the salt bridge interactions at the chain A-chain B interface of the P domain of MNV1 for conformations open and B^a^

Chain A; open conformation	Chain B; closed conformation	MNV1 P domain; open conformation (3lq6) R (Å)	MNV1 P domain; closed conformation (3lqe) R (Å)
GLU338 [OE1]	ARG396 [NH1]	2.56	3.36
GLU338 [OE1]	ARG396 [NH2]	3.29	
GLU338 [OE2]	ARG396 [NH1]	3.48	3.43
GLU338 [OE2]	ARG396 [NH2]		2.66
GLU338 [OE2]	ARG 437 [NH2]	3.01	
ARG396 [NH1]	GLU338 [OE1]	3.38	3.36
ARG396 [NH1]	GLU338 [OE2]	3.60	3.43
ARG396 [NH2]	GLU338 [OE2]	2.41	2.66
ARG396 [NH2]	GLU338 [OE1]	3.63	

a Empty cells represent lack of interactions.

For the MDFF simulations, the hydrogen atoms were added to the starting conformation and a rigid-body fit, with Powell optimization, to the map was performed with the Situs program (70). The CHARMM36 all-atom force field with the CMAP correction (71) was employed with a cutoff distance of 10 Å. The fit is performed *in vacuo* with a scaling factor (ξ) value equal to 0.3 and secondary-structure restraints. A temperature of 300 K was maintained using a Langevin thermostat that was coupled to the heavy atoms with a damping coefficient of 5 ps^−1^. The molecular dynamics simulation was run for a total of 20 ns, with the largest conformational fluctuations occurring within the first 5 ns of the calculation. During the MD simulation, the protein coordinates were saved every picosecond and used to monitor the conformational integrity of the complex. An average RMSD of 1.4 Å was found for the backbone of the conformations of the MD simulation with respect to the assembled PISA model from the cryo-EM reconstruction described earlier. The coordinates saved during the last 2 ns of the MD simulation were used to determine the final average structure shown in Fig. 10.

To investigate the role of explicit solvent in the structural conformation, a second molecular dynamics flexible fitting calculation was performed with explicit solvent and ions. First, the map was trimmed to accommodate one FabD/MNV complex, thereby minimizing the simulation size; second, the system was subjected to solvation with a padding of 20 Å for all sides and ions were added to reproduce a concentration of 0.15 M. The MDFF simulation was performed with the same ξ scaling factor as was used for the *in vacuo* calculations, secondary restraints, and a temperature of 300 K. The presence of the explicit solvent and ions did not result in conformations different from those seen with the *in vacuo* simulations. As *in vacuo* simulations are faster, the subsequent MDFF simulations were performed under those conditions.
