# Supplementary figures and images for: Norovirus Escape from Broadly Neutralizing Antibodies Is Limited to Allostery-Like Mechanisms
Source: mSphere. 2017 Oct 18;2(5):e00334-17. doi: 10.1128/mSphere.00334-17 (PMC5646240; doi:10.1128/mSphere.00334-17)

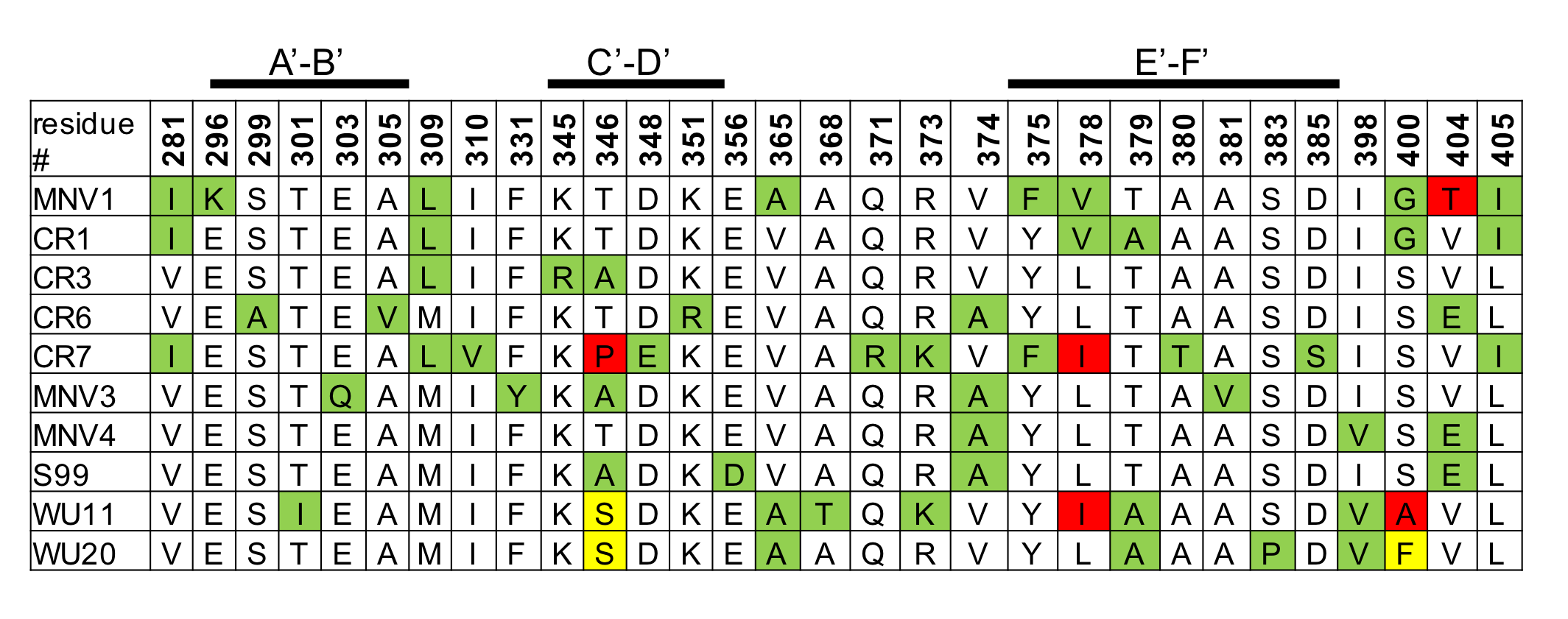

Supplement: FIG S1 [file sph005172386sf1.tif]

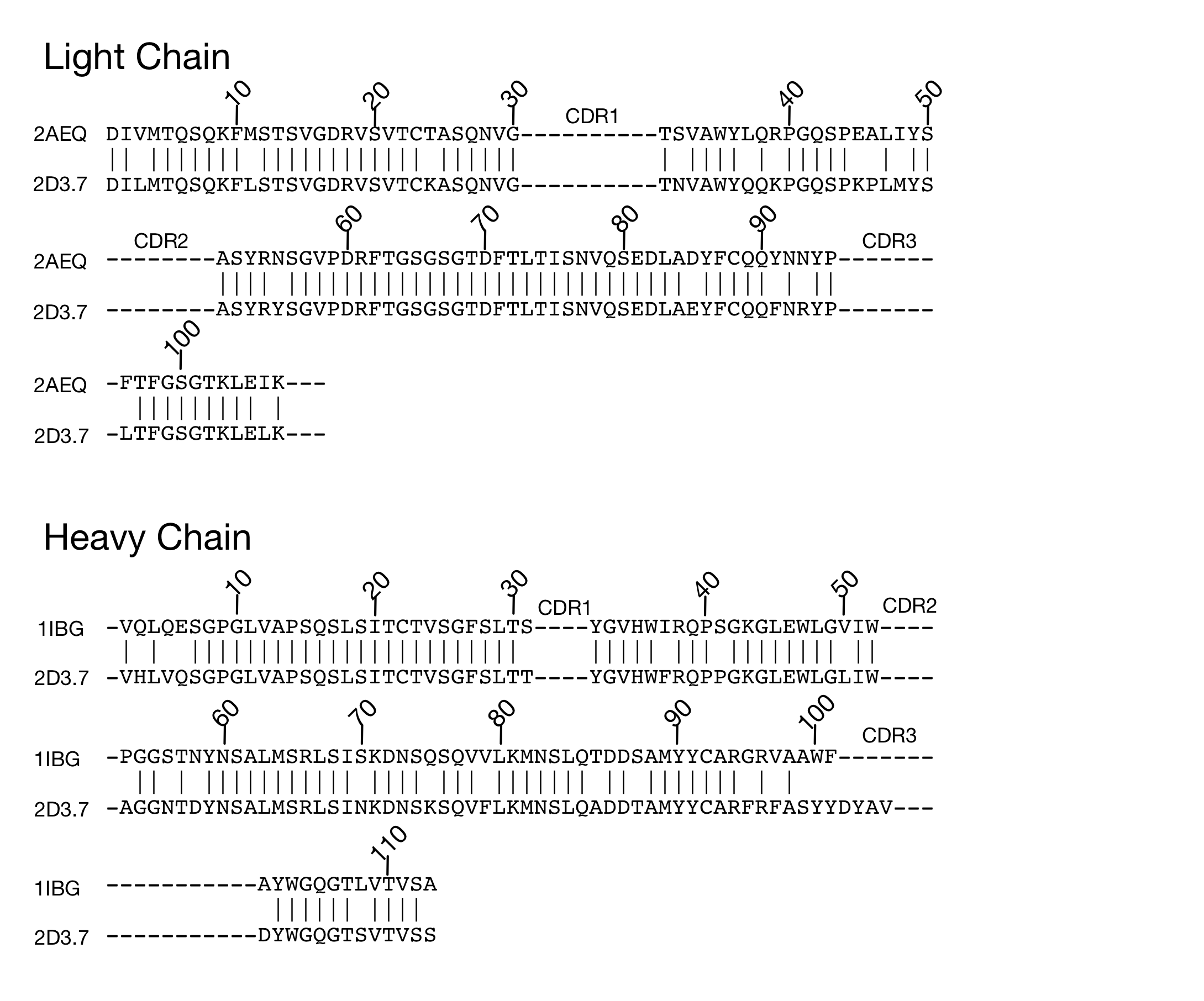

Supplement: FIG S2 [file sph005172386sf2.tif]

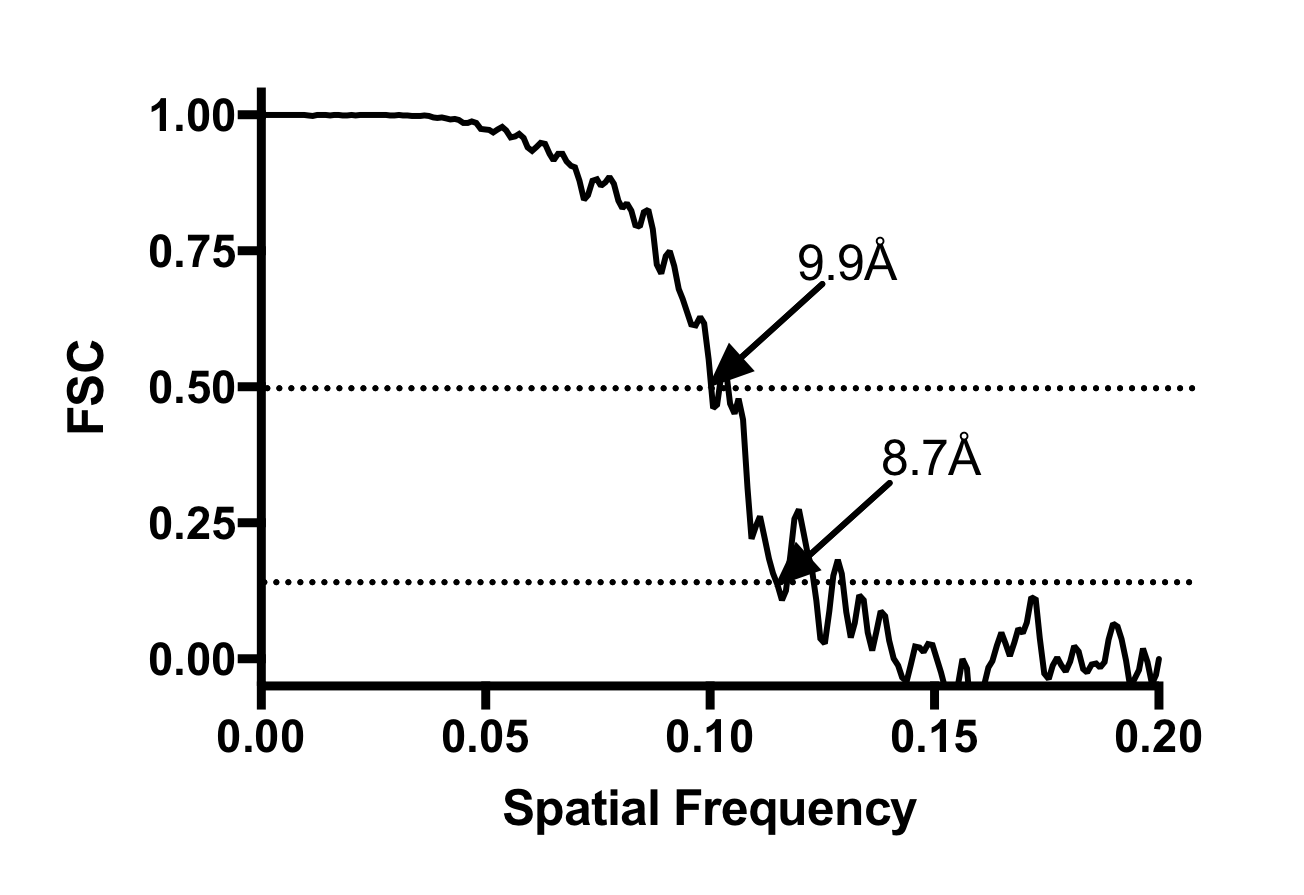

Supplement: FIG S3 [file sph005172386sf3.tif]
